# Transmembrane Mucin Response in Conjunctival Epithelial Cells Exposed to Wall Shear Stresses

**DOI:** 10.3390/ijms24076589

**Published:** 2023-04-01

**Authors:** Shir Itah, David Elad, Ariel J. Jaffa, Dan Grisaru, Mordechai Rosner

**Affiliations:** 1 Department of Biomedical Engineering, Faculty of Engineering, Tel-Aviv University, Tel-Aviv 69978, Israel; 2 Department of Obstetrics and Gynecology, Tel-Aviv University, Tel-Aviv 69978, Israel; 3 Sackler Faculty of Medicine, Tel Aviv University, Tel-Aviv 69978, Israelprof.rosner.m@gmail.com (M.R.); 4 Department of Gynecological Oncology, Lis Maternity Hospital, Tel-Aviv Medical Center, Tel-Aviv 64239, Israel; 5 Department of Ophthalmology, Sheba Medical Center, Ramat-Gan 52620, Israel; 6 Assuta Medical Centers, Tel-Aviv 69710, Israel

**Keywords:** mechanobiology, primary human cells, membrane-tethered mucins, MUC1, MUC16, F-actin, steady airflow

## Abstract

Human conjunctival epithelium cells (HCEC) line the inner surface of the eyelid and cover the sclera and are continuously subjected to wall shear stresses (WSS). The effects of external forces on the conjunctival epithelium are not fully known. The conjunctival epithelium contains stratified squamous cells that synthesize the membrane-spanning mucins MUC1 and MUC16, which play important roles in protecting the ocular surface. Alterations in both gel-forming and membrane-tethered mucins occur in drying ocular surface diseases. The aim of this study was to explore the mechanobiological characteristics of transmembrane mucin secretion and cellular alterations of primary HCEC exposed to airflow-induced WSS perturbations. We exposed the HCEC to a steady WSS of 0.5 dyne/cm^2^ for durations of 15 and 30 min. Cytoskeletal alterations and MUC1 secretions were studied using immunohistochemically fluorescent staining with specific antibodies. We investigated for the first time an in vitro model of membrane-tethered mucin secretion by HCEC in response to WSS. The exposure of HCEC to WSS increased the polymerization of F-actin, altered the cytoskeletal shape and reduced the secretion of membrane-tethered MUC1.

## 1. Introduction

The tear film is the front-line defender of the ocular surface and is composed of an aqueous mucin layer covered by a lipid layer. The aqueous mucin layer contains fluid and soluble factors produced by the lacrimal glands and mucin produced by the goblet cells and transmembrane sources of the human conjunctival epithelial cells (HCEC) [1,2]. The conjunctiva is a thin, semi-transparent, highly vascularized and mucous-secreting tissue that lines the inner surface of the eyelid and curves onto the anterior surface of the eyeball, where it extends to the cornea [3,4]. It contains two major cell types, stratified squamous cells and goblet cells, that discharge different mucins into the tear film. Mucins (MUCs) are high-molecular-weight O-linked glycoproteins and can be divided into gel-forming and membrane-tethered mucins. Stratified squamous cells synthesize the membrane-spanning MUC1, MUC4 and MUC16 that are trafficked to the plasma membranes [5]. In contrast, goblet cells synthesize and secrete the large, gel-forming MUC5AC with a high molecular weight [6].

The membrane-tethered mucins are also known as transmembrane mucins and are characterized by a single membrane-spanning domain, a large extracellular domain and a short cytoplasmic tail. MUC1 is the smallest one and is expressed by both corneal and conjunctival epithelia, and MUC16 is the largest membrane-bound mucin [7]. The membrane-tethered mucins represent a major component of the mucosal glycocalyx of ocular surface epithelial cells [8,9]. The primary functions of the mucins present in the tear film are to maintain the hydration of the ocular surface, to provide lubrication and anti-adhesive properties between the cells of the ocular surface and conjunctiva during blinking and to prevent pathogens from binding to the ocular surface [6,10].

Dry eye is a multifactorial disease of the tears and ocular surface that results in symptoms of discomfort, visual disturbance and tear film instability, with potential damage to the ocular surface. It has been shown that dry eye disease (DED) is associated with changes in mucin expression and glycosylation [11,12]. Several studies have shown that the MUC5AC transcripts in the conjunctival epithelia of patients with DED were significantly lower than those in normal individuals, but not in the expression of MUC1 [12,13,14]. Nevertheless, some investigators hypothesized that mucin expression levels correlate well with DED and may aid clinical evaluations [15]. Changes in mucin (including the transmembrane) expression and glycosylation are important factors in the development and progression of DED. Several studies suggested that transmembrane mucins, such as MUC1, are important regulators of ocular surface homeostasis and inflammation, and that their dysregulation may contribute to the development and progression of DED [16,17]. The loss of goblet cells and the resulting decrease in mucin production is one of the hallmarks of DED, and it can lead to changes in mucin expression and glycosylation in the conjunctiva.

The HCEC are continuously subjected to wall shear stresses (WSS) induced by airflow, eyelid movements, as well as wiping and rubbing of the eyes [10]. Recently, we explored the cytoskeletal alterations and MUC5AC secretion from air–liquid interface (ALI)-cultured primary HCEC after exposure to WSS [18]. In this study, we cultured primary HCEC immersed in medium and investigated the alterations in MUC1 secretion and cytoskeletal modification due to steady-airflow-induced WSS.

## 2. Results

**Figure 1:**
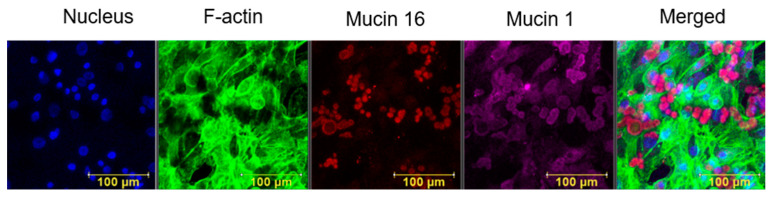
Representative projection of a cultured primary HCEC. Immunofluorescence staining: nucleus (blue), F-actin (green), membrane-tethered Mucin16 (red) and membrane-tethered Mucin1 (purple).

An in vitro model of transmembrane mucin secretion by primary HCEC has been developed for mechanobiological tests. The expression of transmembrane mucins in the in-medium cultured model was validated with specific markers. A representative confocal image of a 250 × 250 μm area after 4 days of culture is depicted in Figure 1. The projection image clearly reveals the presence of MUC1 (purple) and MUC16 (red) in the cultured HCEC. The cells in the sample do not express the same level of mucins and some may not secrete at all.

**Figure 2:**
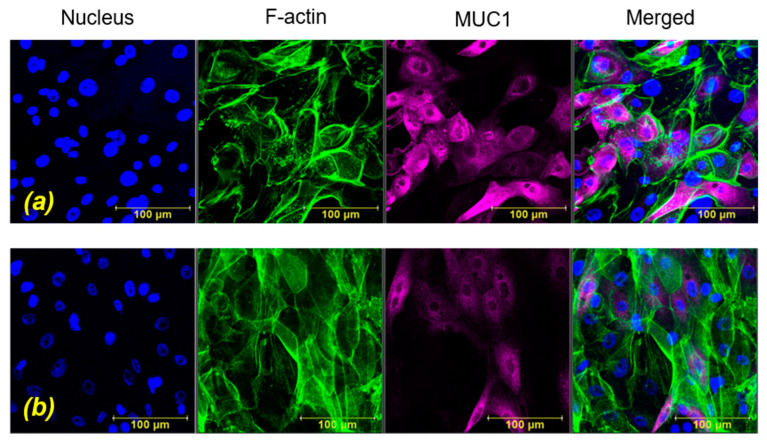
Representative projection images of human conjunctival epithelial cell (HCEC) in vitro model. (**a**) Unstressed control, (**b**) after exposure to wall shear stresses (WSS) of 0.5 dyne/cm^2^ for 15 min. Immunofluorescence staining: nucleus (blue), F-actin (green), MUC1 (purple).

**Figure 3:**
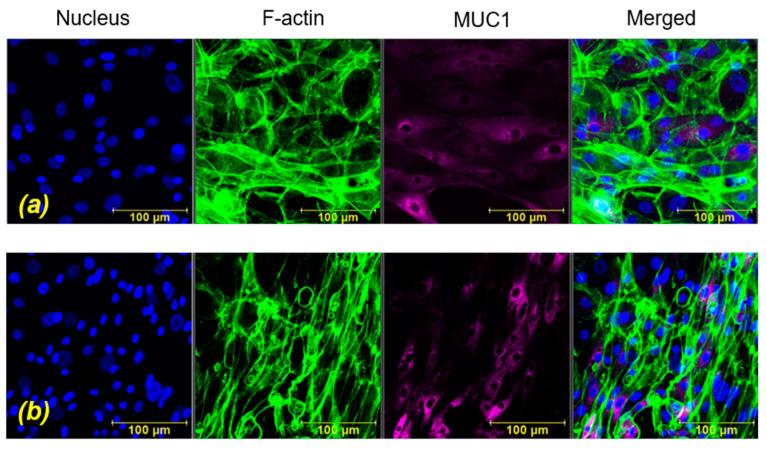
Representative projection images of human conjunctival epithelial cell (HCEC) in vitro model. (**a**) Unstressed control, (**b**) after exposure to wall shear stresses (WSS) of 0.5 dyne/cm^2^ for 30 min. Immunofluorescence staining: nucleus (blue), F-actin (green), MUC1 (purple).

**Figure 4:**
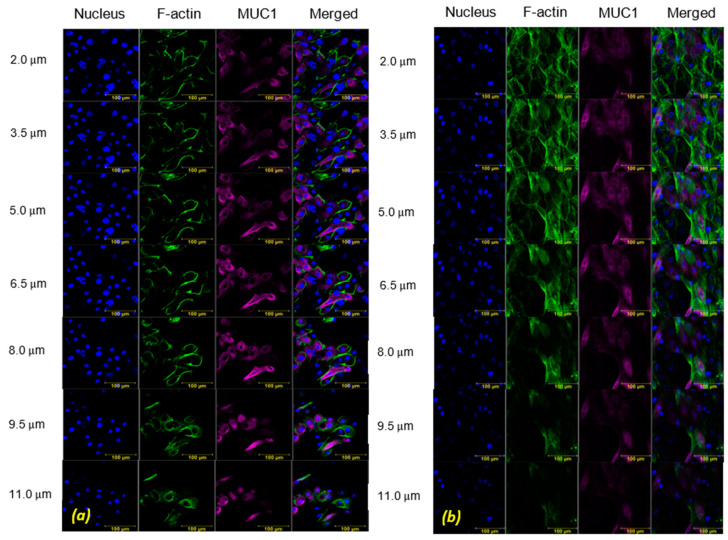
The Z-stack cross-section images of the human conjunctival epithelial cell (HCEC) in vitro model. (**a**) Unstressed control, (**b**) after exposure to wall shear stresses (WSS) of 0.5 dyne/cm^2^ for 15 min. Immunofluorescence staining: nucleus (blue), F-actin (green), MUC1 (purple).

Representative projection images obtained immediately after exposure to WSS for durations of T_15_ and T_30_ are shown in Figure 2 and Figure 3, respectively. These results are shown in comparison to their unexposed controls. Representative cross-section images of the z-stack sections immediately after exposure for a duration of T_15_ and the unexposed controls are shown in Figure 4. Similar images for the duration of T_30_ can be found in Figure S1 in the Supplementary Materials. The cytoskeletal alterations and MUC1 secretion immediately after exposure to WSS were quantified in comparison to the unexposed cultures. It can be observed (especially in Figure 4) that after exposure to WSS, the cells were slightly more elongated compared to the unstressed control and showed increased expression of the intracellular networks of F-actin filaments. Three-dimensional images of the HCEC after exposure to WSS can also be found in Figures S2 and S3 in the Supplementary Materials.

**Figure 5:**
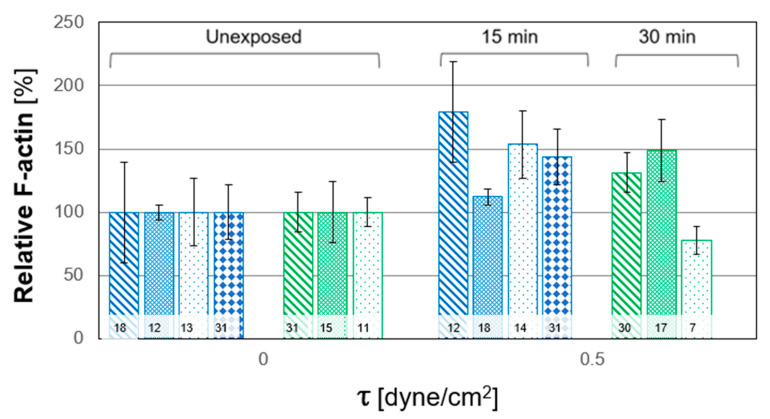
Relative alterations of cytoskeletal F-actin in human conjunctival epithelial cell (HCEC) in vitro model in single tests with HCEC cultured from different subjects. The cultures were exposed to wall shear stresses (WSS) of 0.5 dyne/cm^2^ for durations of 15 min (4 repetitions) and 30 min (3 repetitions). Columns with the same pattern and color represent the control and those after exposure to WSS in a single test. The data obtained after exposure to WSS were scaled by the averaged data for the unexposed control. The values inside the bars denote the number of data points calculated from the sub-regions (n).

**Figure 6:**
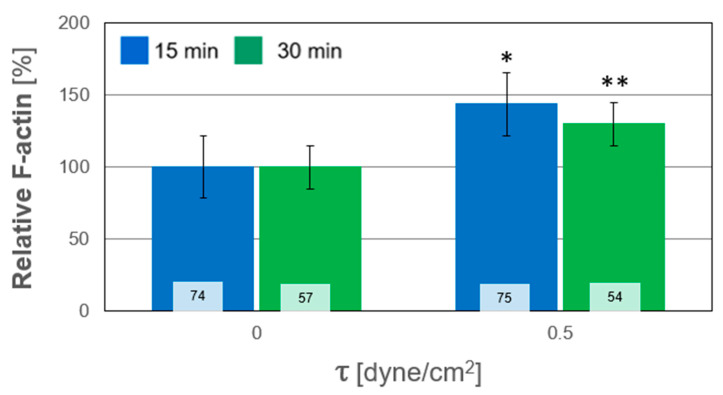
Combined relative alterations of cytoskeletal F-actin in cultured human conjunctival epithelial cells (HCEC) from all tests of exposure to wall shear stresses (WSS) of 0.5 dyne/cm^2^ for durations of 15 min and 30 min. The data obtained after exposure to WSS were scaled by the averaged data for the unexposed controls. * and ** stand for *p* < 0.05, with respect to the unexposed cultures. The values inside the bars denote the number of data points calculated from the sub-regions (n).

**Figure 7:**
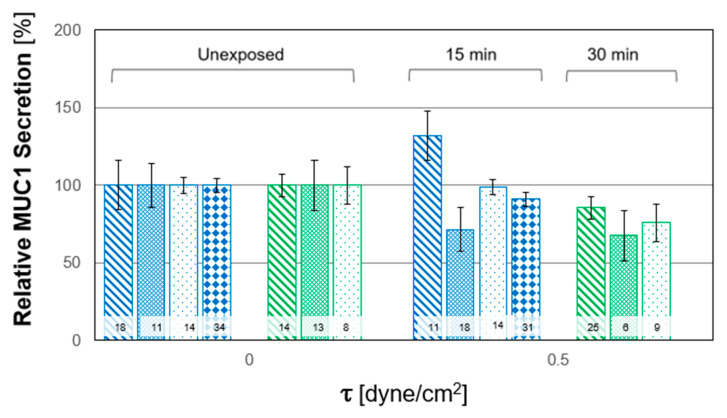
Relative MUC1 secretion measurements in human conjunctival epithelial cell (HCEC) in vitro model in single tests with HCEC cultured from different subjects. The cultures were exposed to wall shear stresses (WSS) of 0.5 dyne/cm^2^ for durations of 15 min (4 repetitions) and 30 min (3 repetitions). Columns with the same pattern and color represent the control and those after exposure to WSS in a single test. The data obtained after exposure to WSS were scaled by the averaged data for the unexposed control. The values inside the bars denote the number of data points calculated from the sub-regions (n).

**Figure 8:**
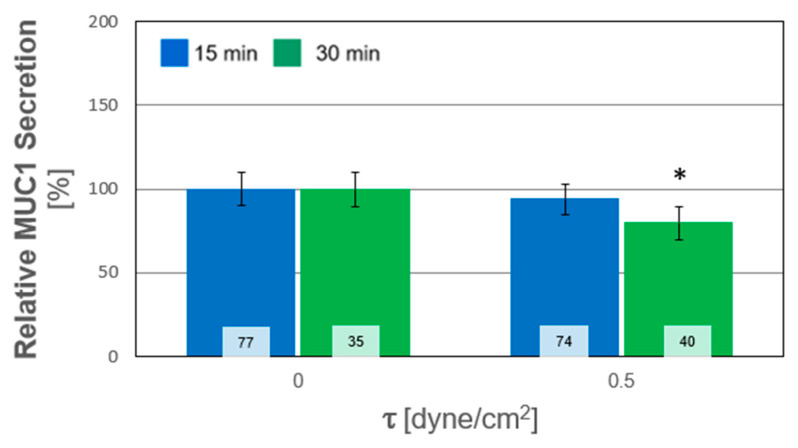
Combined MUC1 secretion measurements in cultured human conjunctival epithelial cells (HCEC) from all tests of exposure to wall shear stresses (WSS) of 0.5 dyne/cm^2^ for durations of 15 min and 30 min. The data obtained after exposure to WSS were scaled by the averaged data for the unexposed controls. * stands for *p* < 0.05, with respect to the static cultures. The values inside the bars denote the number of data points calculated from the sub-regions (n).

In this study, we quantified the relative alterations of F-actin and MUC1 secretion due to WSS from the confocal images. These alterations were quantified from their expression in the Z-stack images relative to that from the averaged unexposed controls. The results of all the experiments for both durations are depicted in Figure 5; each column and pattern represent a single repetition of unstressed and stressed cultures. The combined results of all the exposure tests are summarized in Figure 6. A general trend of increased polymerization of F-actin in response to WSS is clearly observed. The secretion of MUC1 after exposure to WSS was also quantified from their expression in the confocal images relative to the unexposed controls. The results from all the repeated WSS experiments are shown in Figure 7, while the combined results for all the tests of exposure are summarized in Figure 8. The general trend demonstrated a decrease in the expression of MUC1 due to WSS.

## 3. Discussion

The present study is an extension of our previous work in which we examined cytoskeletal alterations and goblet-induced MUC5AC in ALI-cultured HCEC under WSS [18]. Here, we used similar experimental protocols, but cultured the primary HCEC submerged in medium in order to study, for the first time, membrane-tethered MUC1 secretion as well as cytoskeletal F-actin alterations due to exposure to WSS. We exposed the cultured primary HCEC to laminar airflows that induced steady WSS of 0.5 dyne/cm^2^ for durations of 15 and 30 min.

The in vitro model of HCEC demonstrated an adaptive mechanism in response to WSS via modifications in their morphology and actin content, as depicted in Figure 3 and Figure 4. The cells changed their cytoskeletal arrangement and synthesis, which led to a change in the color intensity of F-actin filaments, as they appeared stronger and with an intracellular network of filaments after exposure to WSS, for both exposure times (i.e., T_15_ and T_30_) (i.e., Figure 4). In the post-exposure cultures, F-actin polymerization increased significantly compared to the unexposed controls for both exposure lengths (Figure 6), although the results did not show a significant difference between the exposure durations. The results depicted in Figure 5 demonstrate a similar trend of increased F-actin polymerization compared to the unexposed controls in nearly all the test repetitions for both exposure times. Only for the third repetition with exposure to WSS for a duration of T_30_, an insignificant decrease in F-actin intensity compared to unexposed controls was observed. These results are very consistent with those of the documented observations concerning F-actin polymerization and an increase in stress fibers in epithelial and endothelial cells [18,19,20,21,22,23]. It has been suggested that rapid actin remodeling is a primary mechanism of cellular mechano-transduction [19,24], and the exposure of the HCEC to WSS for short exposure periods of 15 and 30 min supports this assumption. Similarly, the exposure of ALI-cultured HCEC to steady and oscillatory WSS revealed increased F-actin polymerization [18], and the exposure of endothelial cells to steady WSS resulted in alignment in the flow direction due to F-actin reorganization [20].

The expression of F-actin in confocal images was used to analyze the cytoskeleton alterations, since they are the leading components of the CSK that provide mechanical support to the cell and have an important role in the regulation of cell shape and generation of mechanical forces [25]. The results of multiple repetitions with HCEC obtained from different subjects revealed the same trend as shown in Figure 5. Thus, the results of these experiments provide an objective measure of the HCEC response to steady WSS and are not subject-dependent. Moreover, all the experiments were conducted with controlled experimental systems. For example, the measurements of F-actin intensity and mucin secretion were normalized to the number of Z-sections in the specific area, as well as to the number of nuclei in this area, under the assumption that the cell may vanish in response to the airflows and the number of nuclei may be reduced. Thus, the effects of factors other than the steady WSS on the HCEC were reduced. In order to reduce the inter-subject variability, the results were also normalized to the unexposed cultures.

In the present study, we demonstrated the expression of the membrane-tethered MUC1 and MUC16 in in vitro cultures of primary HCEC (Figure 1). Then, we quantified the level of MUC1 secretion in response to steady WSS relatively to that from unexposed controls. The post-exposure values of MUC1 secretion decreased compared to those from the unexposed controls of the same subjects and for both exposure lengths (i.e., T_15_ and T_30_), as depicted in Figure 8. However, this decrease was significant only for the exposure length of T_30_. In addition, the results did not show a significant difference between the two exposure lengths. Since the exposure to WSS was relatively short, we assumed that the pre-exposure secreted MUC1 in the cultured HCEC was partially washed away during the exposure to airflow, without further production during the experiments. Nevertheless, changes in mucin secretion, including the membrane-tethered mucins, are one of the mechanisms associated with the development and progression of DED [16,17].

In previous studies, similar trends of a decrease in the secretion of MUC5AC were reported, although not significant [14,15,16,18], and were explained by the relatively small amount of goblet cells in the HCEC [18]. Furthermore, it is known that the membrane-tethered mucins are smaller in molecular weight than their large, polymeric, secreted mucin counterparts, such as MUC5AC. MUC1 is the smallest of the three ocular surface transmembrane mucins (e.g., 120–300 kDa) and is expressed by the corneal and conjunctival epithelia [7], as well as by the lacrimal gland and lacrimal duct epithelia [26,27]. Some studies proposed a correlation between inflammatory mediators in the tears (e.g., IL-6) that downregulate the levels of MUC1 and dry eye disease [28]. Accordingly, it has been assumed that alterations in the membrane-tethered mucins may play a role in dry eye disease. The expression of MUC1 in the human stratified corneal and conjunctival epithelium was previously demonstrated in ex vivo models via the RT-PCR technique [7].

## 4. Materials and Methods

### 4.1. Cells Isolation and Culture

We used in-vitro-cultured primary HCEC, which are the most physiologically relevant model. The protocol for HCEC isolation and culture was similar to that in our previous article [18], and here we briefly describe the methodology. The primary HCEC were isolated from conjunctival specimens from the upper fornix, over the Muller muscle, that were obtained during the surgical correction of ptosis from patients with healthy conjunctiva. The study was approved by the IRB committees of the Sheba (6943-20-SMC) and Assuta Medical Centers (ASMC 0011-18) and the donors signed consent forms. In total, we harvested HCEC from 12 patients: 10 females (age range 50 to 77) and 2 males (aged 31 and 83). The specimens were from either of one or both eyelids from different subjects. For each test, the HCEC harvested from the eyelid tissue of both eyes of one or two patients were used to ensure sufficient cells for a complete WSS test and control.

The cells were dissociated by incubation in 1.2 U/mL Dispase II (17105-041, Gibco, Waltham, MA, USA) solution at 37 °C for 1–2 h, and then the loosened cells were scraped, washed and centrifuged at 1100 RPM for 5 min. Then, we suspended the cells in a serum-free hormone bronchial epithelial growth medium (BEGM) (CC-3171, Lonza, Walkersville, MD, USA). The medium was supplemented with 0.15 mg/mL bovine serum albumin (BSA) (A4919, Sigma-Aldrich, St. Louis, MO, USA) and a SingleQuots supplement pack (CC-4175, Lonza) containing insulin, hydrocortisone, epinephrine, triiodothyronine, transferrin, retinoic acid, bovine pituitary extract, gentamicin, amphotericin and human epidermal growth factor. The cells were plated in BEGM within a collagen-type-I-coated 25 cm^2^ flask and incubated in a 5% CO^2^ incubator at 37 °C. After 24 h, the cells were washed with Dulbecco’s phosphate-buffered saline without calcium and magnesium (DPBS w/o salt; Biological Industries, Beit Haemek, Israel) and fed with new BEGM. The medium was changed every 2–3 days till the HCEC reached 80–90% confluence, usually within 7–8 days.

At this stage, the cells were trypsinized, counted and transferred for further culture on a synthetic hydrophilic membrane (PolyTetraFluoroEthylene (PTFE) 0.1 μm porous, JVWP02500, Merck, Cork, Ireland) in custom-designed wells [18,29]. These wells have an effective area of 0.8 cm^2^ for cell culture and can be disassembled for installation of the cultured cells in a flow chamber. The PTFE membrane was coated with 16 μg/100 μL of cell attachment matrix (Entactin–Collagen IV–Laminin (ECL); 08-110, Merck) diluted in serum-free Dulbecco′s modified Eagle′s medium high-glucose (DMEM-H) (D6429, Sigma-Aldrich) to a final volume of 100 μL and incubated for 1 h at 37 °C. The cells were seeded at a density of 1.5–1.6 × 10^5^ cells per well and cultured while submerged in a medium of 50:50 BEGM and DMEM-H (Sigma-Aldrich), with the epidermal growth factor reduced 20-fold from its amount in the supplement pack (i.e., 25 µL). After 4–5 days of culture while submerged in medium, the in vitro model of HCEC was ready for exposure to WSS experiments.

### 4.2. Setup for Exposure of HCEC to Wall Shear Stresses

The isolated HCEC were cultured in the custom-designed wells, which could be disassembled into a cylindrical medium holder and well bottom. The well bottom with the cultured HCEC was installed in a flow chamber for the application of WSS on the apical surfaces of the cells [29]. The flow chamber was an 18-cm-long rectangular conduit with a 20 × 10 mm cross-section that could hold 3 well bottoms with the cultured HCEC co-planar with the bottom plane of the WSS field [29]. The steady unidirectional airflow experiments were conducted in a laminar flow hood in a room environment (e.g., 25 ± 2 °C and 40–50% RH), with the flow chamber connected to a steady air source via a flowmeter (Rotameter FL-3440ST, Omega Engineering Inc., Norwalk, CN, USA). A 2-m-long silicon tube connected the flow chamber to the air source to ensure a fully developed airflow within the flow chamber. The exit was also connected to a silicon tube to prevent exit disturbances.

### 4.3. Experimental Protocol

The protocol for experiments was similar to our previous work with HCEC [18]. The experiments were performed with passage 2 of primary HCEC cultured while submerged in medium for 4–5 days. The well components were sterilized in an autoclave before seeding the HCEC. The flow chamber, tubes and all working tools were cleaned with ethanol 70%. The HCEC were exposed to steady airflows of 30 L/min (i.e., Re = 1961) that induced WSS of τ = 0.5 dyne/cm^2^ on top of the cells for two durations, T_15_ = 15 min and T_30_ = 30 min. These durations are based on our previous experience with nasal and conjunctiva epithelial cells exposed to WSS [16,17]. For each test of either of the durations, we used 6 well bottoms with HCEC: 3 were placed in the flow chamber and 3 unexposed controls were placed in the hood for the whole experiment. Each test was repeated at least 3 times. All 6 wells for a single experiment were harvested from 1 or 2 subjects; 2 sets were from 1 subject and 5 were from two subjects. In total, we harvested HCEC from 12 patients. All the WSS experiments with a duration of T_15_ were repeated 4 times, while all the WSS experiments with a duration of T_30_ were repeated 3 times. After the experiments, all the wells were fixed for confocal imaging quantitative analyses.

### 4.4. Immunofluorescence Staining

Immunofluorescence stains were used for model validation and the quantitative evaluation of cytoskeletal F-actin alterations and MUC1 secretion. The following specific antibodies were used (i) 1:500 dilution of phalloidin-iFluor 488 (Ab 176753, Abcam, Cambridge, UK), (ii) 1:50 dilution of anti-MUC1 (VU4H5) Alexa Fluor^®^ 680 antibody (sc-7313 AF680, ENCO, Petah Tikva, Israel), (iii) 1:50 dilution of anti-MUC16 (C-6) Alexa Fluor^®^ 594 (sc-365002 AF594, ENCO, Petah Tikva, Israel) and (iv) DAPI (D9542, Abcam) for the nuclei. The HCEC controls and those obtained immediately after exposure to WSS were fixed by incubation in 4% paraformaldehyde (PFA) for 10 min at RT. The cells were then incubated overnight at 4 °C with DPBS 1% BSA and the specific antibodies. The HCEC were washed three times with DPBS for 5 min after each step in the shaker machine. Following staining, the cultured cells on the well bottom were imaged under a Leica SP8 confocal microscope with a 20×/0.75 dry lens.

### 4.5. Relative Quantification of F-Actin Alterations and MUC1 Secretion

The cytoskeletal F-actin alterations and MUC1 secretion in response to exposure to WSS were quantified from their corresponding expression in the Z-stack slices of confocal images, using a methodology that we developed in our lab [18]. Since the fluorescence emission is the outcome of binding between the color marker and the molecules, we assumed that the amount of the reflected intensity of the fluorophore color may be correlated with the mass of stained molecules [19]. For this purpose, we used the LAS-X (Leica) and randomly selected 3–4 regions of 775 × 775 μm (i.e., 1024 × 1024 pixels) from the confocal images of each well. Then, we selected smaller sub-regions of 100 × 100 μm (i.e., 132 × 132 pixels) for the analysis of the F-actin and MUC1 expression. All the confocal images from all 6 wells of a single repetition of each duration (i.e., exposed to WSS and non-exposed controls) were acquired in a single session. In order to identify the Z-stack cross-sections of each layer of the HCEC, we conducted a manual search. The levels of intensity for F-actin (Phalloidin, Green) and MUC1 (Magenta) were quantified using a MATLAB algorithm by summing all the pixels in each Z-slice. The values of intensity from all sub-regions for each exposure were averaged and scaled with respect to the average of the non-exposed control. The results for each sub-region of 100 × 100 μm provided one data point for the analysis.

### 4.6. Statistical Analysis

The quantitative results were expressed as mean ± standard error (i.e., standard deviation/√n). Sampled values of mean ± 2.1 of the standard deviation were discarded. For each test, we had 3 wells of unexposed control cells that were averaged and used for comparison. Significance levels were calculated using the Microsoft Excel software, version 2302. A two-tailed independent-samples *t*-test was used to determine statistical significance. The *p* value of less than 0.05 was considered statistically significant.

## 5. Conclusions

In conclusion, we investigated for the first time an in vitro model of membrane-tethered mucin secretion by in-medium cultured primary HCEC that were exposed to steady-airflow-induced WSS. The results confirmed that the membrane-tethered MUC1 is secreted and expressed by HCEC. In addition, the results for WSS at both exposure lengths revealed similar trends regarding cytoskeletal alterations and membrane-tethered mucin secretion.

## Data Availability

The data used to support the findings of this study are available from the corresponding author upon request.

## Supplementary Materials

The following supporting information can be downloaded at: https://www.mdpi.com/article/10.3390/ijms24076589/s1.
