# Supplementary material for: Transmembrane Mucin Response in Conjunctival Epithelial Cells Exposed to Wall Shear Stresses
Source: Int J Mol Sci. 2023 Apr 1;24(7):6589. doi: 10.3390/ijms24076589 (PMC10095083; doi:10.3390/ijms24076589)
Supplement: Supplementary file 1 [file ijms-24-06589-s001.zip › ijms-2211387-supplementary.pdf]

Supplementary Material

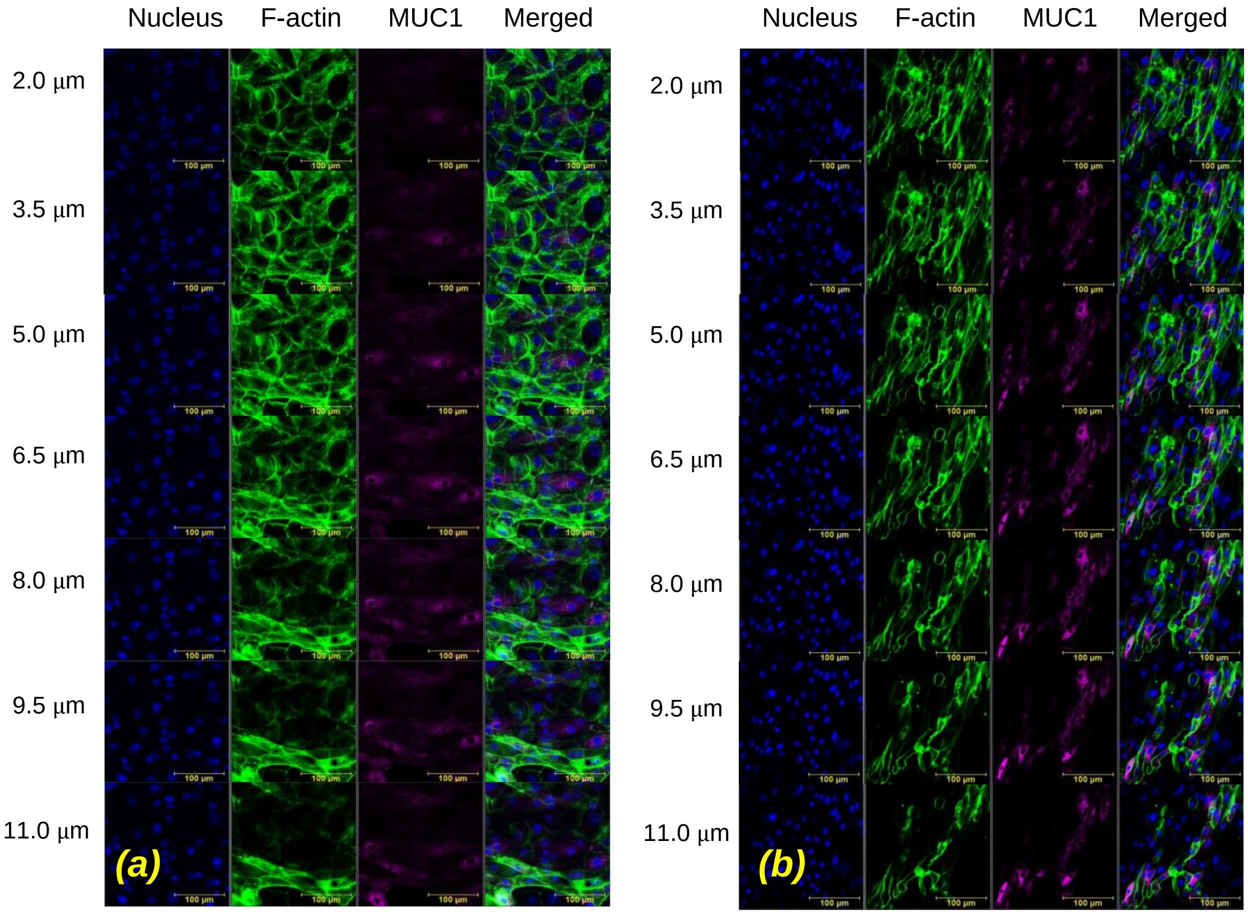

Figure S1. The Z-Stack cross-section images of the human conjunctival epithelial cells (HCEC) *in vitro* model. a) Unstressed control, b) Post exposure to wall shear stresses (WSS) of 0.5 dyne/cm^2 for 30 min. Immunofluorescence staining: nucleus (blue), F-actin (green), MUC1 (purple).

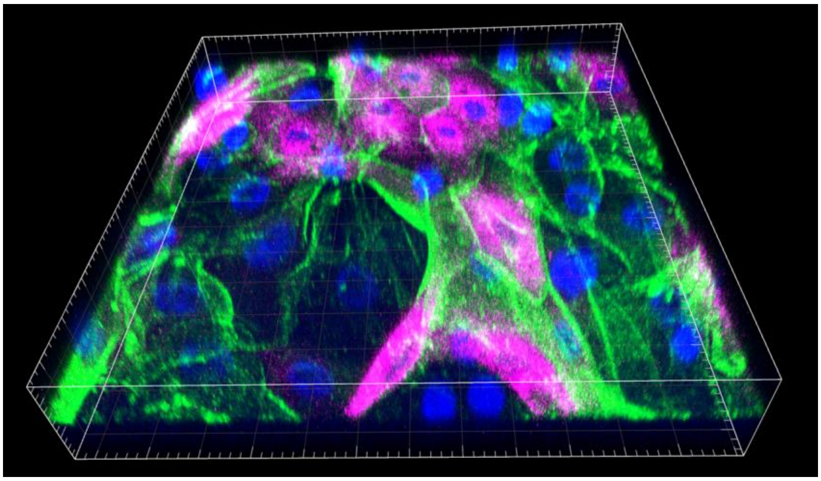

Figure S2. Three-dimensional image of the human conjunctival epithelial cells (HCEC) *in vitro* model after exposure to wall shear stresses (WSS) of 0.5 dyne/cm^2 for 15 min. Immunofluorescence staining: nucleus (blue), F-actin (green), MUC1 (purple).

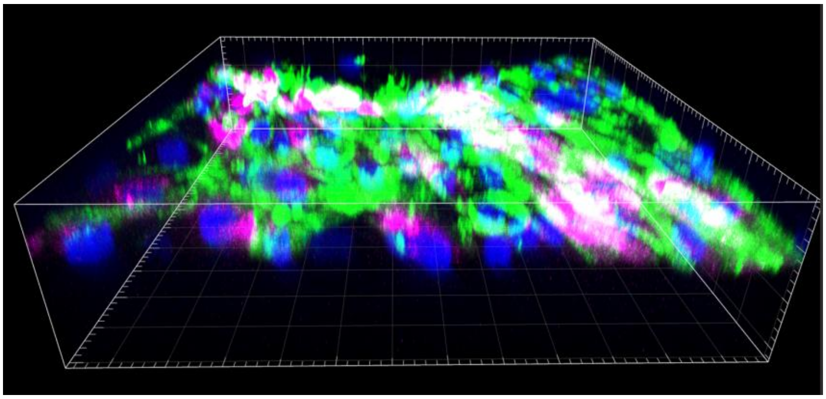

Figure S3. Three-dimensional image of the human conjunctival epithelial cells (HCEC) *in vitro* model after exposure to wall shear stresses (WSS) of 0.5 dyne/cm^2 for 30 min. Immunofluorescence staining: nucleus (blue), F-actin (green), MUC1 (purple).
